# C1q/TNF-Related Protein 9 Attenuates Atherosclerosis by Inhibiting Hyperglycemia-Induced Endothelial Cell Senescence Through the AMPKα/KLF4 Signaling Pathway

**DOI:** 10.3389/fphar.2021.758792

**Published:** 2021-10-22

**Authors:** Gang Wang, Baihe Han, Ruoxi Zhang, Qi Liu, Xuedong Wang, Xingtao Huang, Dandan Liu, Weishen Qiao, Mengyue Yang, Xing Luo, Jingbo Hou, Bo Yu

**Affiliations:** ^1^ The Key Laboratory of Myocardial Ischemia Organization, Chinese Ministry of Education, Harbin, China; ^2^ Department of Cardiology Organization, The Second Affiliated Hospital of Harbin Medical University, Harbin, China; ^3^ Department of Cardiology, Harbin Yinghua Hospital, Harbin, China

**Keywords:** atherosclerosis, senescence, hyperglycemia, AMPK, KLF4, CTRP9

## Abstract

Hyperglycemia-induced endothelial cell senescence has been widely reported to be involved in the pathogenesis of type 2 diabetes mellitus‒accelerated atherosclerosis. Thus, understanding the underlying mechanisms and identifying potential therapeutic targets for endothelial cell senescence are valuable for attenuating atherosclerosis progression. C1q/tumor necrosis factor-related protein 9 (CTRP9), an emerging potential cardiokine, exerts a significant protective effect with respect to atherosclerosis, particularly in endothelial cells. However, the exact mechanism by which CTRP9 prevents endothelial cells from hyperglycemia-induced senescence remains unclear. This study aimed to investigate the effects of CTRP9 on hyperglycemia-induced endothelial cell senescence and atherosclerotic plaque formation in diabetic apolipoprotein E knockout (ApoE KO) mice. Human umbilical vein endothelial cells (HUVECs) were cultured in normal glucose (5.5 mM) and high glucose (40 mM) with or without recombinant human CTRP9 protein (3 μg/ml) for 48 h. Purified lentiviruses overexpressing CTRP9 (Lv-CTRP9) and control vectors containing green fluorescent protein (Lv-GFP) were injected via the tail vein into streptozotocin-induced diabetic ApoE KO mice. Results revealed that exposure of HUVECs to HG significantly increased the expression of Krüppel-like factor 4 (KLF4) and cyclin-dependent kinase inhibitor p21 (p21) and decreased that of telomerase reverse transcriptase (TERT). Treatment with recombinant human CTRP9 protein protected HUVECs from HG-induced premature senescence and dysfunction. CTRP9 promoted the phosphorylation of AMP-activated kinase (AMPK), attenuated the expression of KLF4 and p21 induced by HG, and increased the expression of TERT in HUVECs. Furthermore, in the background of AMPKα knockdown or KLF4 activation, the protective effects of CTRP9 were abolished. *In-vivo* experiments showed that the overexpression of CTRP9 inhibited vascular senescence and reduced atherosclerotic plaque formation in ApoE KO mice with diabetes**.** In conclusion, we demonstrate that KLF4 upregulation plays a crucial role in HG-induced endothelial senescence. This anti-atherosclerotic effect of CTRP9 may be partly attributed to the inhibition of HG-induced endothelial senescence through an AMPKα/KLF4-dependent mechanism, suggesting that CTRP9 could benefit further therapeutic approaches for type 2 diabetes mellitus‒accelerated atherosclerosis.

## Introduction

Diabetes is associated with a high incidence of atherosclerotic coronary artery disease, resulting in premature mortality (Einarson et al., 2018). Although the underlying pathogenesis involves many factors, data from human and animal studies have supported a direct proatherogenic role of endothelial premature senescence induced by hyperglycemia (Minamino et al., 2002; Bornfeldt and Tabas, 2011; Stojanović et al., 2020). Endothelial senescence indicates a permanent arrest of cell growth and proliferation. Pathological studies have shown that senescent endothelial cells accumulate in the subendothelial space at the onset of atherosclerosis, where they drive disease pathogenesis by increasing the expression of key atherogenic inflammatory cytokines and chemokines, resulting in the generation of a phenotype referred to as the senescence-associated secretory phenotype (SASP) (Childs et al., 2016; Childs et al., 2018). Furthermore, a number of *in-vitro* and *in-vivo* studies have shown that endothelial cell senescence compromises endothelial barrier function owing to the disruption of cell proliferation, permeability, and motility, possibly contributing to endothelial erosion and intraplaque hemorrhage (Lähteenvuo and Rosenzweig, 2012; Boyle et al., 2017; Sedding et al., 2018). Moreover, senescent endothelial cells promote endothelial dysfunction by increasing reactive oxygen species (ROS) levels and decreasing nitric oxide (NO) production (Tian and Li, 2014). Therefore, endothelial senescence plays a prominent role in the initiation and progression of atherosclerotic lesions. Progress in preventing atherosclerosis has been stalled by the epidemic of diabetes. Understanding the mechanisms by which hyperglycemia promotes endothelial cell senescence and the clinical application of anti-endothelial senescence strategies might provde avenues for the development of new therapeutic strategies to prevent and treat atherosclerotic vascular disease.

CTRP9—an adipokine as well as a cardiokine—is the closest adiponectin paralog. Extensive studies have found that CTRP9 exerts a significant protective effect with respect to atherosclerosis, involving multiple factors and pathways (Yang et al., 2017). CTRP9 maintains a beneficial lipid profile and promotes plaque stability in addition to ameliorating endothelial dysfunction, mitigating inflammatory responses, inhibits the transformation of vascular smooth muscle cells into macrophage-like cells, and reducing foam cell formation (Liu et al., 2017; Sun et al., 2017; Zhang et al., 2018). Notably, recent studies indicate that treatment with CTRP9, an emerging potential anti-senescence molecule, inhibited cell senescence via the AMPK signaling pathway (Li et al., 2018; Rezabakhsh et al., 2020). KLF4 is a zinc-finger-containing transcription factor that plays a crucial role in maintaining cell senescence in response to cellular stress. Studies have shown that KLF4 typically upregulates p21 to promote cell growth arrest (Chew et al., 2011). Moreover, KLF4 could negatively modulate TERT to influence telomere length; short telomere length induces cellular senescence (Minamino et al., 2002; Torrance and Goldband, 2020). AMPKα can negatively regulate KLF4 expression in advanced atherosclerotic plaques in the brachiocephalic arteries (Ding et al., 2016). However, whether AMPKα and KLF4 expression is critical for mediating the beneficial effects of CTRP9 in high glucose (HG)-induced endothelial cell senescence has not yet been clearly demonstrated. This study aimed to determine the effects and mechanisms of action of CTRP9 in HG-induced endothelial cell senescence, and to elucidate whether CTRP9 inhibits the progression of atherosclerotic plaques through anti-senescence properties *in vivo*.

## Materials and Methods

### Cell Culture and Treatment

Human umbilical vein endothelial cells (HUVECs) were obtained from Science Cell Research Laboratories (Carlsbad, CA, United States) and cultured in Dulbecco’s Modified Eagle Medium (DMEM, GE Healthcare Life Science HyClone Laboratories, Logan, Utah, United States) supplemented with 1% endothelial cell growth factors, 5% fetal bovine serum, and 1% penicillin/streptomycin at 37°C in a humidified atmosphere containing 5% CO_2_ until the start of the experiment. D-Glucose (G8150) and D-mannitol (M8140) were obtained from Solarbio (Beijing, China). For the studies comparing the effects of mannitol, HUVECs were incubated with different media that were defined as NG (glucose concentration of 5.5 mM), HG (40 mM) (Arunachalam et al., 2014; Wu et al., 2021), osmotic control (40 mM: 5.5 mM of glucose +34.5 mM of D-mannitol) for 48 h. The media were changed every 24 h.

Recombinant human CTRP9 (6537-TN, R&D Systems) was reconstituted in sterile phosphate buffer saline (PBS) at a concentration of 250 μg/ml. After synchronization, the HUVECs were incubated with NG or HG medium containing CTRP9 at a concentration of 3 μg/ml (Liu et al., 2017; Zhang et al., 2018) for 48 h. APTO (HY-16291, MedChem Express) was used as an inducer of KLF4, and HUVECs were treated with vehicle or APTO at a concentration of 5 μM for 24 h to evaluate the involvement of KLF4 in HG-induced senescence.

### Human Umbilical Vein Endothelial Cell Synchronization

It is considered that high-purity G0/G1 phase cells can be obtained by culturing the HUVECs in completely serum-free culture media at 37°C in a humidified atmosphere containing 5% CO_2_ for 12 h; this provides a reliable experimental basis for subsequent experimental research.

### Staining for Senescence-Associated β-Galactosidase (SA-β-Gal)

The HUVECs (4 × 10^4^ cells/well) were first plated in 12-well plates. After the necessary stimulation, they were fixed and washed three times with PBS. Then, they were incubated with the SA-β-gal staining reagent (C0602, Beyotime Biotechnology, China) and maintained at 37°C overnight. The number of SA-β-gal-positive cells (blue staining) was counted under a fluorescence microscope (DMI4000B; Leica, Wetzlar, Germany).

### Measurement of ROS Levels

The HUVECs (2 × 10^4^ cells/well) were first plated in 24-well plates. After the necessary stimulation, the accumulation of cellular ROS was detected using a Reactive Oxygen Species assay kit (S0033S, Beyotime Biotechnology, China). Then, they were treated with 2’,7’-Dichlorodi-hydrofluorescein diacetate (DCFH-DA, 10 μM) in the dark for 20 min at 37°C; they were then washed with serum-free medium three times. Fluorescence was observed using a fluorescence microscope (DMI4000B; Leica, Wetzlar, Germany).

### Measurement of Total NO Levels

The HUVECs (2 × 10^4^ cells/well) were first plated in 24-well plates. After the necessary stimulation, total NO production in the culture medium was determined by measuring the concentration of nitrate and nitrite, a stable metabolite of NO, through the modified Griess reaction method. The procedure was performed according to the protocol stipulated by the manufacturer of the Total Nitric Oxide assay kit (S0021S, Beyotime Biotechnology, China).

### siRNA Transfection

Small interfering RNAs (siRNAs) specific to AMPKα, KLF4, and the negative control were synthesized by RiboBio (Guangzhou, China). The HUVECs were first plated in 12-well plates (1 × 10^5^ cells/well); upon reaching 40% confluence, the cells were transfected with 50 μM siRNA using the riboFECT™ CP Reagent (RiboBio, Guangzhou, China), according to the manufacturer’s instructions. After 48 h of incubation with the siRNAs, the HUVECs were exposed to HG (40 mM) medium for 48 h in the presence or absence of CTRP9 (3 μg/ml) and then collected for quantitative real-time PCR and western blotting analyses.

### RNA Isolation and RT-PCR

Total RNA was isolated from the HUVECs (2 × 10^6^ cells/well) using the TRIZOL reagent (15596018, Invitrogen, CA, United States) according to the manufacturer’s instructions. First-strand cDNA was synthesized from 1 μg of total RNA using the iScript gDNA Clear cDNA Synthesis Kit (170–8890, Bio-Rad Laboratories, Redmond, United States). Quantitative RT-PCR was performed using a Fast EvaGreen Supermix (172-5260, Bio-Rad Laboratories) on a CFX96 Real-Time PCR Detection System (Bio-Rad Laboratories) following the manufacturer’s protocol. The following optimized PCR conditions were used: 95°C for 30 s, 95°C for 5 s, and 40 cycles at 60°C for 5 s. The mRNA levels of the target genes were normalized to the endogenous GAPDH levels, and the expression levels of the target genes were analyzed using the 2^−ΔΔ^ct method. Table 1 presents all the primers used for qRT-PCR.

**TABLE 1:** Primers for qRT-PCR.

Gene	Forward	Reverse
AMPKα	5′-ACC​AAG​GGC​ACG​CCA​TAC-3′	5′-TCT​TCC​TTC​GTA​CAC​GCA​AA-3′
KLF4	5′-GGG​AGA​AGA​CAC​TGC​GTC​A-3	5′-GGA​AGC​ACT​GGG​GGA​AGT-3′
VCAM-1	5′-CAG​TAA​GGC​AGG​CTG​TAA​AAG​A-3′	5′-TGG​AGC​TGG​TAG​ACC​CTC​G-3′
IL-6	5′-ACT​CAC​CTC​TTC​AGA​ACG​AAT​TG-3′	5′-CCA​TCT​TTG​GAA​GGT​TCA​GGT​TG-3′
IL-8	5′-ACT​GAG​AGT​GAT​TGA​GAG​TGG​AC-3′	5′-AAC​CCT​CTG​CAC​CCA​GTT​TTC-3′
TNF-α	5′-GAG​TGA​CAA​GCC​TGT​AGC​CC-3′	5′-GCA​ATG​ATC​CCA​AAG​TAG​ACC-3′
GAPDH	5′-ACG​GAT​TTG​GTC​GTA​TTG​GGC	5′-TTG​ACG​GTG​CCA​TGG​AAT​TTG-3′

### Western Blotting Analysis

Protein extracts from the HUVECs (2 × 10^6^ cells/well) were separated by 10% sodium dodecyl sulfate-polyacrylamide gel electrophoresis and transferred onto polyvinylidene fluoride membranes (ISEQ00010, Millipore, Billerica, MA, United States) using a semidry transblot apparatus (Bio-Rad Laboratories, Redmond, United States). Subsequently, the membranes were blocked with 5% nonfat dried milk (R&D Systems, Minneapolis, MN, United States) in Tris-buffered saline-Tween 20 (TBST) for 1 h at room temperature and then probed with specific primary mouse or rabbit antibodies against KLF4 (Abcam, Cambridge, MA, United States, Cat. No.: ab215036), AMPK (Cell Signaling Technology (CST), Danvers, MA, United States, Cat. No.: 2532S), p-AMPK (CST, Cat. No.:2535S), p21 (Abcam, Cat. No.: ab109520), TERT (Abcam, Cat. No.: ab32020), and β-actin (TA-09, Zhongshanjinqiao, Inc., Beijing, China) overnight at 4°C. The membranes were then washed with TBST thrice and incubated with the peroxidase-conjugated second antibody (ZB-2301/ZB-2305, Zhongshanjinqiao) for 1 h at room temperature. The immunoreactive bands were detected by chemiluminescence methods and visualized using the Luminescent Imaging Workstation (Tanon, Shanghai, China; 6,600); the relative intensities of the bands were measured and analyzed using the Image J software.

### Immunofluorescence Assay

To detect the activation of KLF4, HUVECs (1 × 10^4^ cells/well) were fixed and permeated at room temperature; then, they were incubated with primary antibodies against KLF4 (diluted in goat serum, 1:100) overnight at 4°C. Next, the cells were washed with TBST and incubated with fluorescent secondary antibody diluted with goat serum (1: 500) at room temperature for 60 min in the dark. Finally, 2-(4-amidinophenyl)-6-indolecarbamidine (DAPI) was used to stain the cell nuclei for 10 min. The cells were viewed and photographed on a confocal laser microscope at 100×/×200 magnification (LSM 800, ZEISS, Germany).

### Animal Model

Seven-week-old male ApoE KO mice (weighing 19–21 g, C57BL/6J background) were randomly divided into four mice per cage, with access to high-fat diet (HFD) containing 0.25% cholesterol and 15% cocoa butter for 16 weeks until sacrifice. The animal grouping and timeline of the experimental protocol are shown in Figure 6. As described previously, diabetic ApoE KO mice were constructed via the intraperitoneal injection of streptozotocin (STZ), which was diluted with citrate buffer (pH 4.5; final concentration, 1%), at a dose of 50 mg/kg/day for five consecutive days after they were fed with a HFD for 4 weeks (Sun et al., 2015). ApoE KO mice (*n* = 8) injected with the vehicle served as the non-DM control. Only the mice with continuous blood glucose levels >15 mmol/L were recruited to the DM group (*n* = 24). These diabetic mice were then randomly divided into three groups: DM + normal saline (NS) group (n = 8), DM + Lv-CTRP9 (lentivirus expressing the CTRP9, *n* = 8), DM + Lv-GFP (null lentivirus expressing GFP, *n* = 8). Lv-CTRP9 and Lv-GFP, diluted to a total volume of 100 μL (2 × 10^7^ TU/mouse), were injected into the tail vein of each mouse from the DM + Lv-CTRP9 and DM + Lv-GFP groups, respectively; the mice injected with NS served as the vehicle controls. Then, all the mice in the group were fed with HFD until the end of the 17th week. All animal care-associated protocols were conducted in accordance with the “Principles of Animal Care” (Ethical and Animal Welfare Committee of Heilongjiang Province, China) and were approved by the ethics review board of the Harbin Medical University (SYDW 2019-253).

### Histological Analysis

Eight weeks after the treatments, all the mice were euthanized for the subsequent studies. The whole aorta was rapidly removed and washed in PBS. Half of the aortas was fixed in 4% neutral formaldehyde for histological analysis, and the other half was frozen and stored in liquid nitrogen for molecular studies. The aortic arches were used for SA-β-gal staining. The roots of the aortas were embedded in optimal cutting temperature compound and 7-μm-thick cryosections were prepared for hematoxylin-eosin (HE) staining and immunofluorescence analysis. The lesion areas of the aortas and aortic roots were analyzed using the Image J software.

### Statistical Analysis

All statistical analyses were performed using the GraphPad Prism 8.0 software (GraphPad Software, San Diego, CA, United States) and were presented as the means ± standard deviations (SDs). Differences among groups were determined using a one-way ANOVA test. Each experiment was repeated at least three times, and differences with *p* values <0.05 were considered statistically significant.

## Results

### Hyperglycemia Promotes Human Umbilical Vein Endothelial Cell Senescence by Influencing the Expression of KLF4 and Downstream Signaling Targets

**FIGURE 1:**
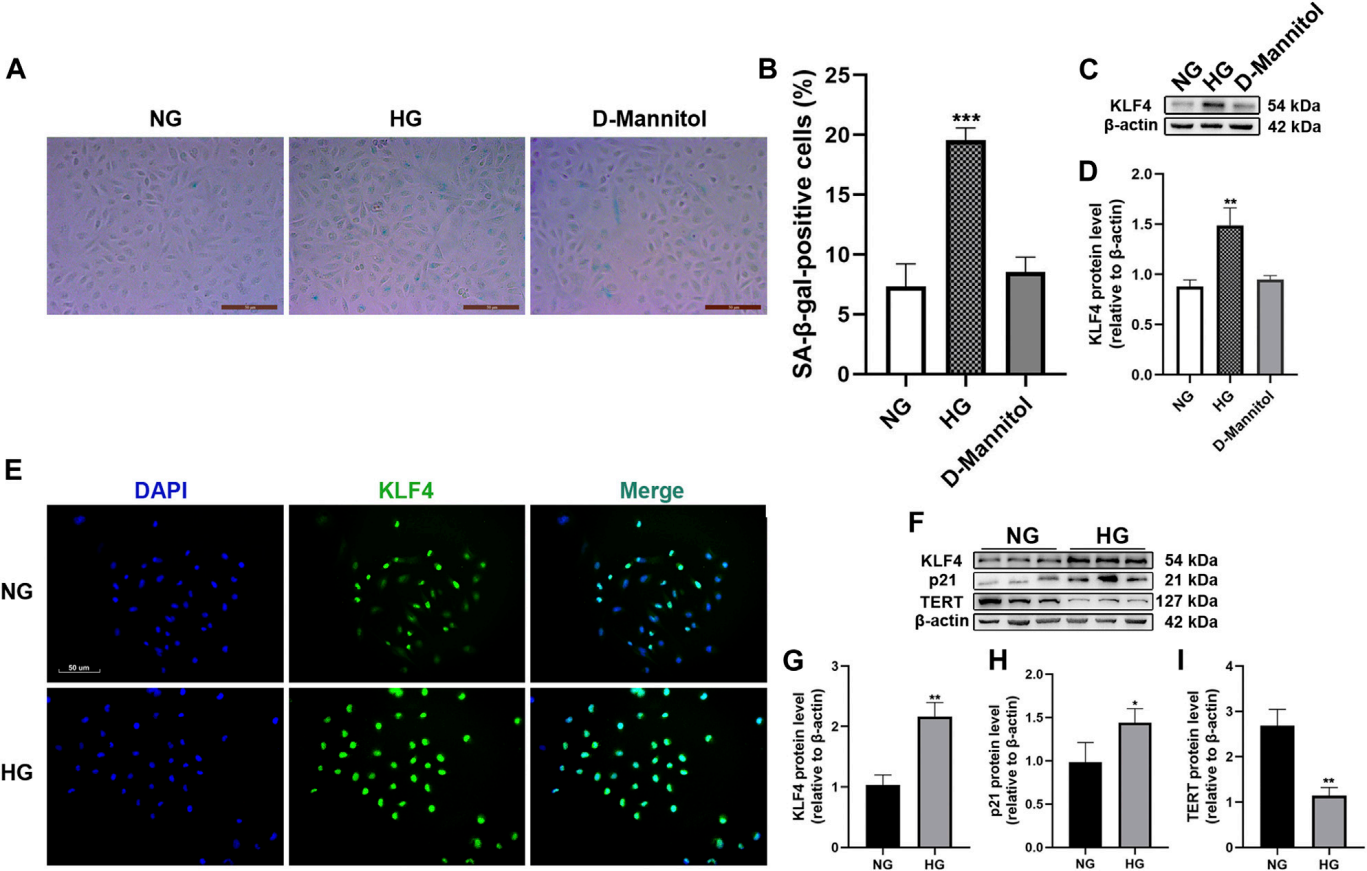
Effect of HG exposure on KLF4, p21, and TERT expression in HUVECs. **(A)** For the studies comparing the effects of mannitol, HUVECs were incubated with media consisting of NG or HG, and D-mannitol for 48 h. Cells were fixed and stained for SA-β-gal activity. The images are taken by ×10magnification. **(B)** Histogram represents the percentage of SA-β-gal-positive cells per microscopic field. **(C)** Cell lysates were used to determine the KLF4 protein levels. **(D)** Results were normalized to controls, and histograms represent the relative intensity of KLF4. Values represent mean ± SEM (*n* = 3–4 per group). **p < 0.01, ***p < 0.001, significantly different from NG. **(E)** HUVECs were also cultured either in NG or HG media for 48 h, and intracellular KLF4 levels were measured by immunofluorescence staining. The images were taken at ×100 magnification and sections were stained with KLF4 (green) and DAPI (blue). A representative image from three separate experiments is illustrated. **(F)** KLF4, p21, and TERT protein levels were determined by immunoblotting**. (G**–**I)** Results were normalized to controls, and histograms represent the relative intensity of KLF4, p21, and TERT. Values represent mean ± SEM (*n* = 3–4 per group). **p < 0.01, ***p < 0.001, significantly different from NG.

The effects of HG on HUVEC senescence were determined using SA-β-gal staining. The percentage of SA-β-gal-positive cells increased after exposure to HG for 48 h (*p* < 0.001, Figures 1A,B). The effect of HG on KLF4 expression in HUVECs was determined by immunoblotting. HG treatment for 48 h resulted in a significant increase in KLF4 expression compared to NG treatment (*p* < 0.01, Figures 1C,D). However, exposure of HUVECs to D-mannitol (osmotic control) for 48 h did not result in an increase in SA-β-gal-positive cells or KLF4 expression compared to the observations in HUVECs maintained in NG (*p* > 0.05). Immunofluorescence revealed increased KLF4 expression in HG-treated HUVECs (Figure 1E). The expression of p21 and TERT, the downstream targets of KLF4 and important mediators of senescence, was determined by immunoblotting (Figure 1F). In the HG-treatment group, an increase in KLF4 expression (*p* < 0.01, Figure 1G) was accompanied by a significant increase in p21 expression and decrease in TERT expression compared to that in the NG-treatment group (*p* < 0.05 and <0.01, Figures 1H,I). The results indicated that upregulation of KLF4 is associated with increased p21 expression and decreased TERT expression in HUVECs exposed to HG for 48 h.

### Hyperglycemia-Induced Human Umbilical Vein Endothelial Cell Senescence is KLF4 Dependent

**FIGURE 2:**
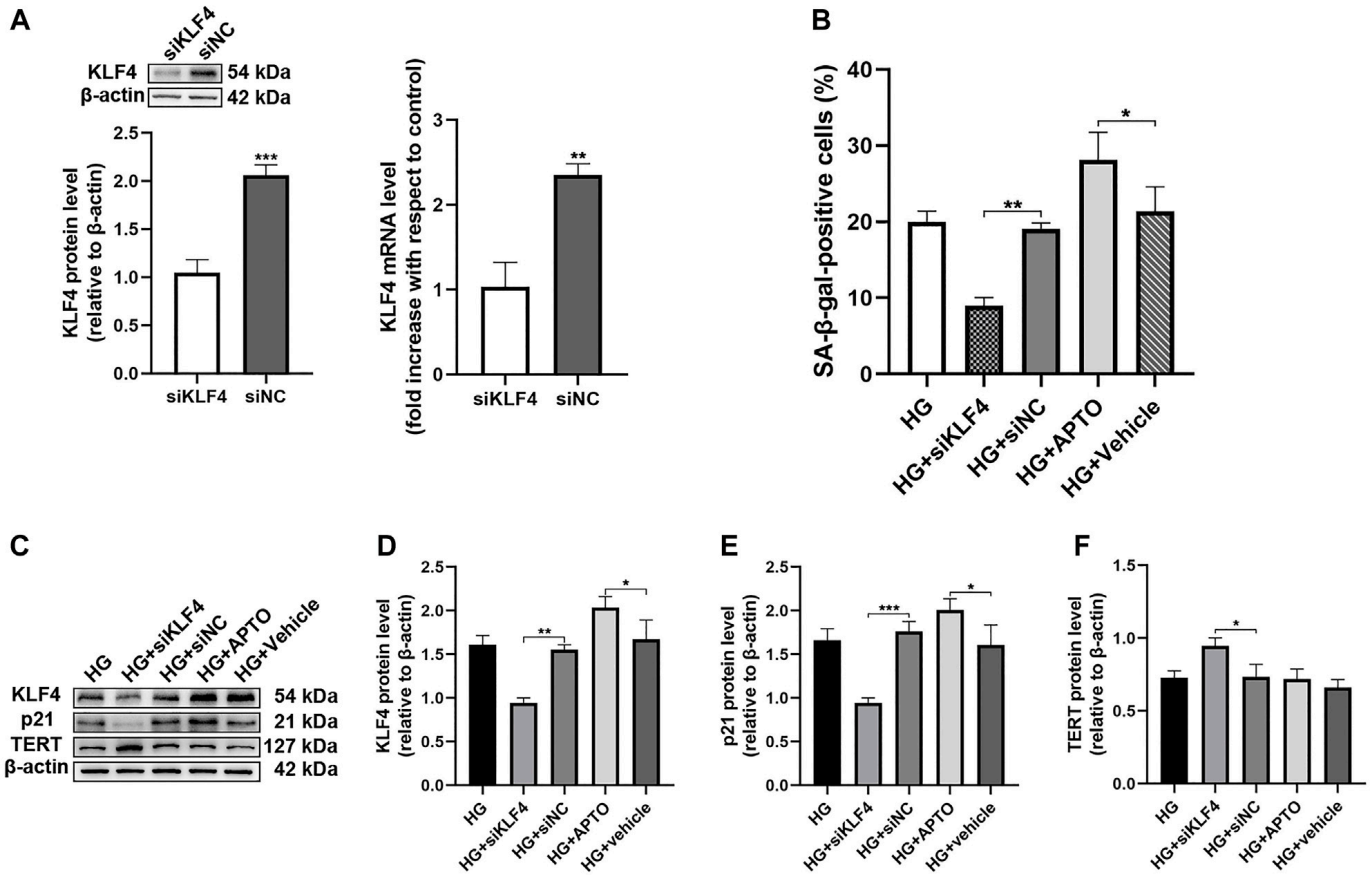
Hyperglycemia-induced HUVEC senescence is KLF4 dependent. **(A)** KLF4, as measured by immunoblotting and qRT-PCR in HUVECs after transfection with siNC or siKLF4 for 48 h. **(B)** Cells were fixed and stained for SA-β-gal activity and the histogram represents the percentage of SA-β-gal-positive cells per microscopic field. Values represent mean ± SEM. *p < 0.05, **p < 0.01. **(C)** KLF4, p21, and TERT protein levels were determined by immunoblotting. **(D–F)** Results were normalized to controls, and histograms represent the relative intensity of KLF4, p21, and TERT. Values represent mean ± SEM (*n* = 3–4 per group). *p < 0.05, **p < 0.01, ***p < 0.001.

To further assess the effects of KLF4 on senescence, KLF4 was induced and knocked down using APTO and siKLF4 in HUVECs (Figure 2A), respectively. KLF4 induction using APTO results in G1 arrest *in vitro* (Local et al., 2018). Our results showed that siRNA-mediated KLF4 knockdown in HUVECs resulted in a reduced number of SA-β-gal-positive cells along with a significant downregulation of p21 (*p* < 0.01 and <0.001, Figures 2B,E). Furthermore, a significant increase in the expression of the anti-senescence protein TERT was observed in HUVECs treated with HG and siKLF4 compared to those treated with HG and siNC (*p* < 0.05, Figure 2F). However, HG-treated HUVECs exposed to APTO exhibited more SA-β-gal-positive cells (*p* < 0.05, Figure 2B) and a significant upregulation of KLF4 and p21 compared to HG-treated HUVECs exposed to vehicle (both *p* < 0.05, Figures 2D,E). The results indicated that siRNA-mediated KLF4 downregulation attenuated senescence and that pharmacological overexpression of KLF4 was enhanced in the background of HG treatment. Taken together, these findings indicate that KLF4 expression was positively correlated with HG-induced senescence in HUVECs.

### C1q/Tumor Necrosis Factor-Related Protein 9 Mitigates HG-Induced Endothelial Cell Senescence, Senescence-Sssociated Secretory Phenotype, and Endothelial Dysfunction

**FIGURE 3:**
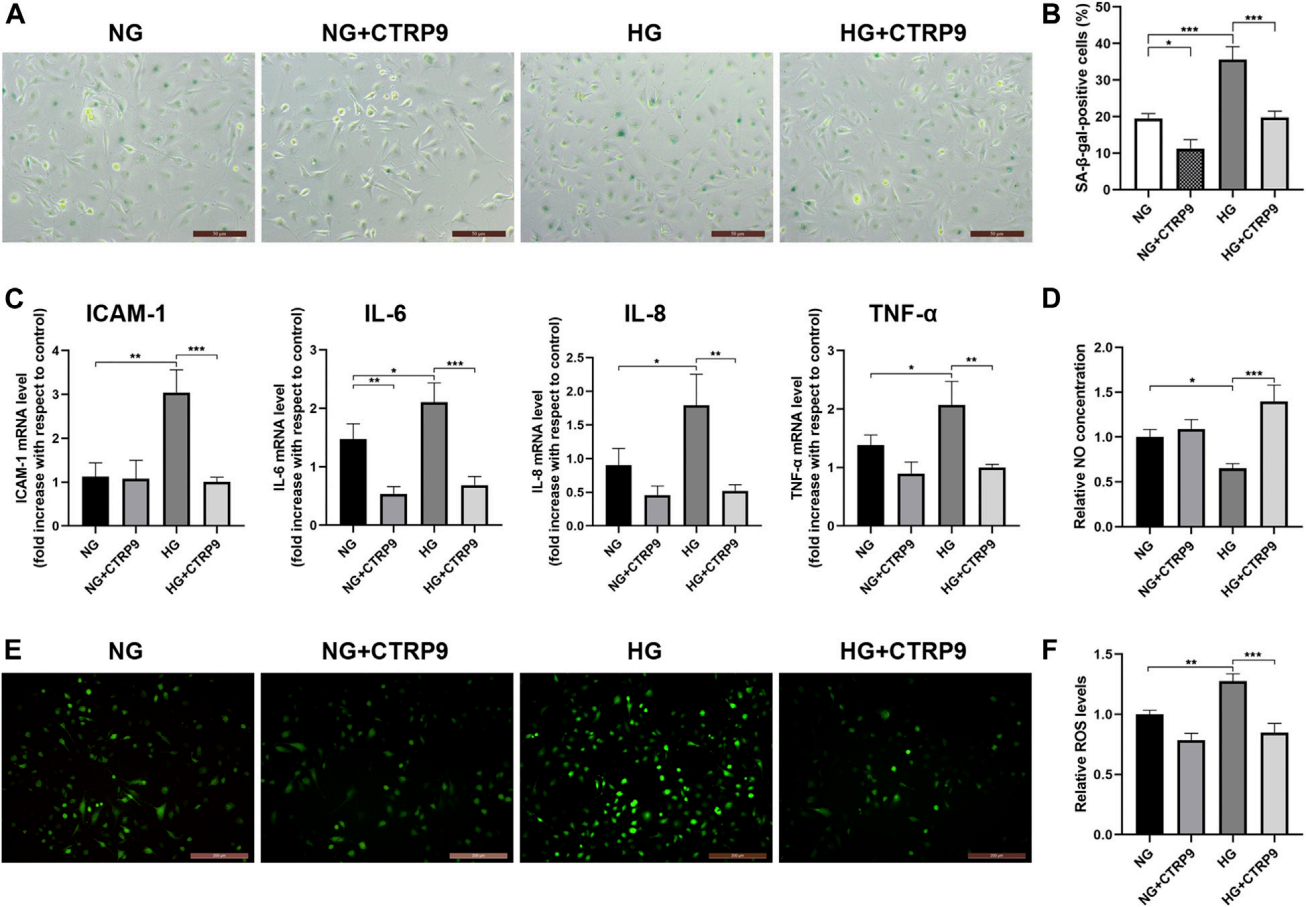
CTRP9 alleviates HG-induced senescence, SASP, and HUVEC dysfunction. **(A)** Cells were fixed and stained for SA-β-gal activity. The images are taken by ×10magnification. **(B)** Histogram represents the quantitative analysis of SA-β-gal-positive cells per microscopic field**. (C)** Cell lysates were used to detect several key SASP component cytokine mRNA levels by qRT-PCR. Results were normalized to controls, and histograms represent the relative intensity of IACM-1, IL-6, IL-8, and TNF-α levels. **(D)** Total NO production in the culture medium was determined by the modified Griess reaction method, and the histogram represents the relative intensity of NO. **(E)** Cells were fixed and stained with dihydroethidium to detect intracellular ROS levels. The images were taken at ×100magnification. **(F)** The histogram represents the relative intensity of fluorescence signal per microscopic field. Values represent mean ± SEM (*n* = 3–4 per group). *p < 0.05, **p < 0.01, ***p < 0.001.

Experiments were designed to demonstrate the protective effect of CTRP9 against HG-induced HUVEC senescence. HUVECs were maintained in media containing either NG or HG, with or without CTRP9. The number of SA-β-gal-positive cells was higher in HG-treated HUVECs than in NG-treated HUVECs (*p* < 0.001, Figures 3A,B). Nevertheless, there was evidence of senescence in NG-treated HUVECs, suggesting that the effects of glucose were concentration-dependent. However, CTRP9-treated HUVECs cultured in HG exhibited reduced endothelial senescence compared to the untreated HUVECs cultured in HG (*p* < 0.001, Figures 3A,B). The SASP—largely comprising proinflammatory cytokines and chemokines—has been identified as an important player and key therapeutic target in atherosclerosis. HG increased the transcript-level expression of several key SASP components, including ICAM-1, IL-6, IL-8, and TNF-α in HUVECs (all *p* < 0.05, Figure 3C). CTRP9 reduced the transcript-level expression of these SASP components in HG-treated HUVECs (all *p* < 0.01, Figure 3C).

Endothelial cell dysfunction is a hallmark of vascular disease and is characterized by reduced NO bioavailability and enhanced ROS production. Total NO levels were analyzed and found to be significantly lowered following culture in HG medium for 48 h (*p* < 0.05, Figure 3D). CTRP9 increased the NO levels in HG-treated HUVECs (*p* < 0.001, Figure 3D). Next, HUVECs were stained with dihydroethidium for measuring ROS levels. Intracellular ROS production increased in HG-treated HUVECs compared to that in NG-treated HUVECs as evidenced by ROS-induced nuclear fluorescence (*p* < 0.01, Figures 3E,F). However, CTRP9 treatment of HUVECs cultured in HG resulted in lower levels of ROS (*p* < 0.001, Figures 3E,F). Collectively, these findings showed that CTRP9 inhibits HUVEC senescence, SASP, and endothelial dysfunction.

### C1q/Tumor Necrosis Factor-Related Protein 9-Mediated Anti-Senescence Effect is Mediated via the AMPKα/KLF4 Pathway

**FIGURE 4:**
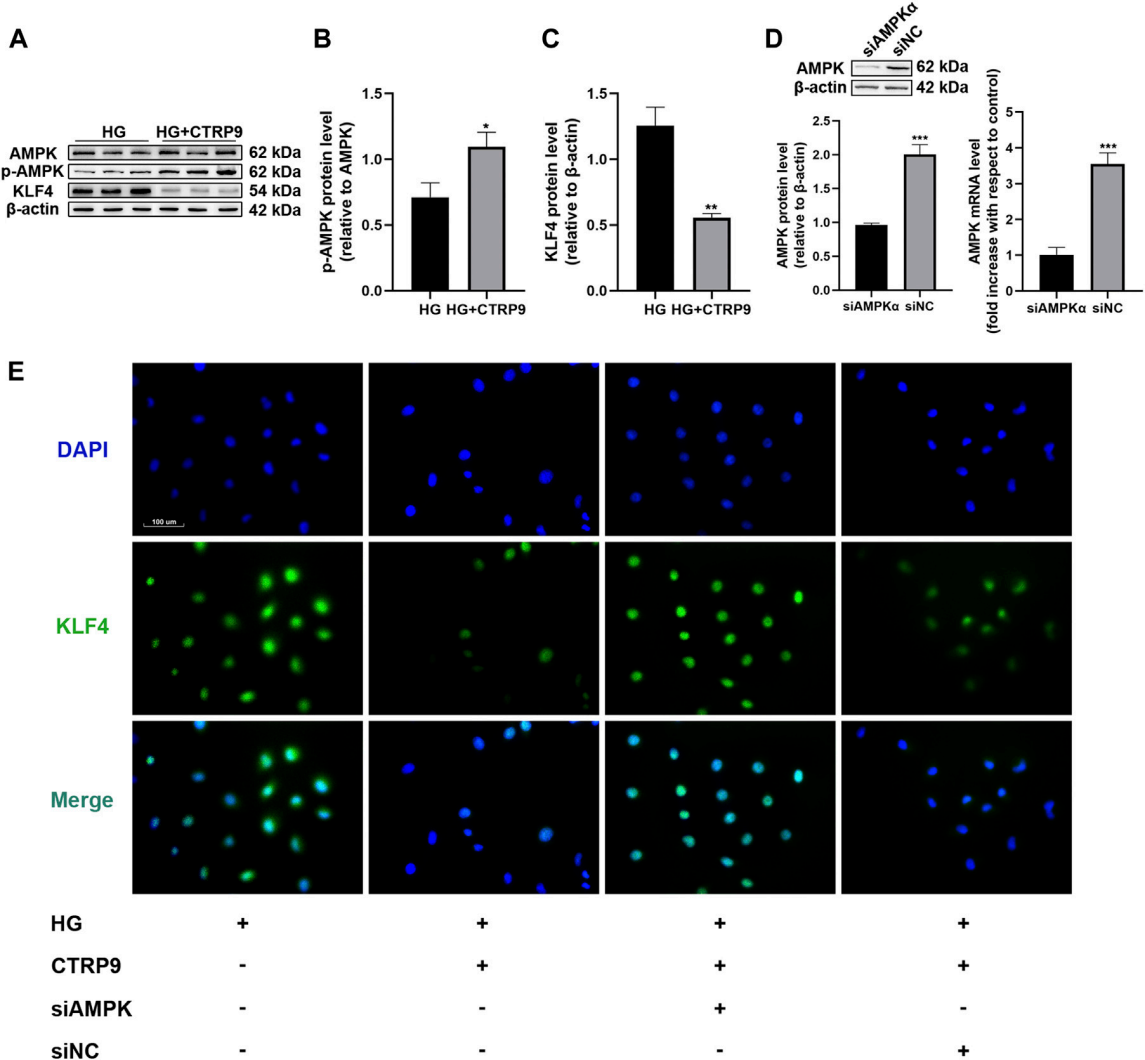
CTRP9 treatment promotes the phosphorylation of AMPK and inhibits KLF4 protein expression. **(A)** AMPK, p-AMPK and KLF4 protein levels were determined by immunoblotting. **(B–C)** Results were normalized to controls, and histograms represent the relative intensity of KLF4 and p-AMPK. Values represent mean ± SEM (*n* = 3-4 per group). *p < 0.05, **p < 0.01, significantly different from HG. **(D)** AMPKα, as measured by immunoblotting, and qRT-PCR results of HUVECs after transfection with siNC or siAMPKα for 48 h. Values represent mean ± SEM (*n* = 3–4 per group). ***p < 0.001. **(E)** Intracellular KLF4 levels were measured by immunofluorescence staining. The images were taken at ×200magnification and sections were stained with KLF4 (green) and DAPI (blue). A representative image from three separate experiments is illustrated.

To assess the cellular and molecular basis of CTRP9-associated decrease in HUVEC senescence, we first examined AMPKα and KLF4 expression in HUVECs treated with HG and CTRP9 for 48 h. There was a significant reduction in KLF4 expression and induction of p-AMPK when HUVECs were maintained in HG and treated with CTRP9 (*p* < 0.05 and <0.01, Figures 4B,C). We then downregulated AMPKα expression in HUVECs using specific siRNA (Figure 4D). Immunofluorescence analysis showed that CTRP9-treated HUVECs cultured in HG exhibited decreased KLF4 expression, and that AMPKα knockdown reversed this effect (Figure 4E).

**FIGURE 5:**
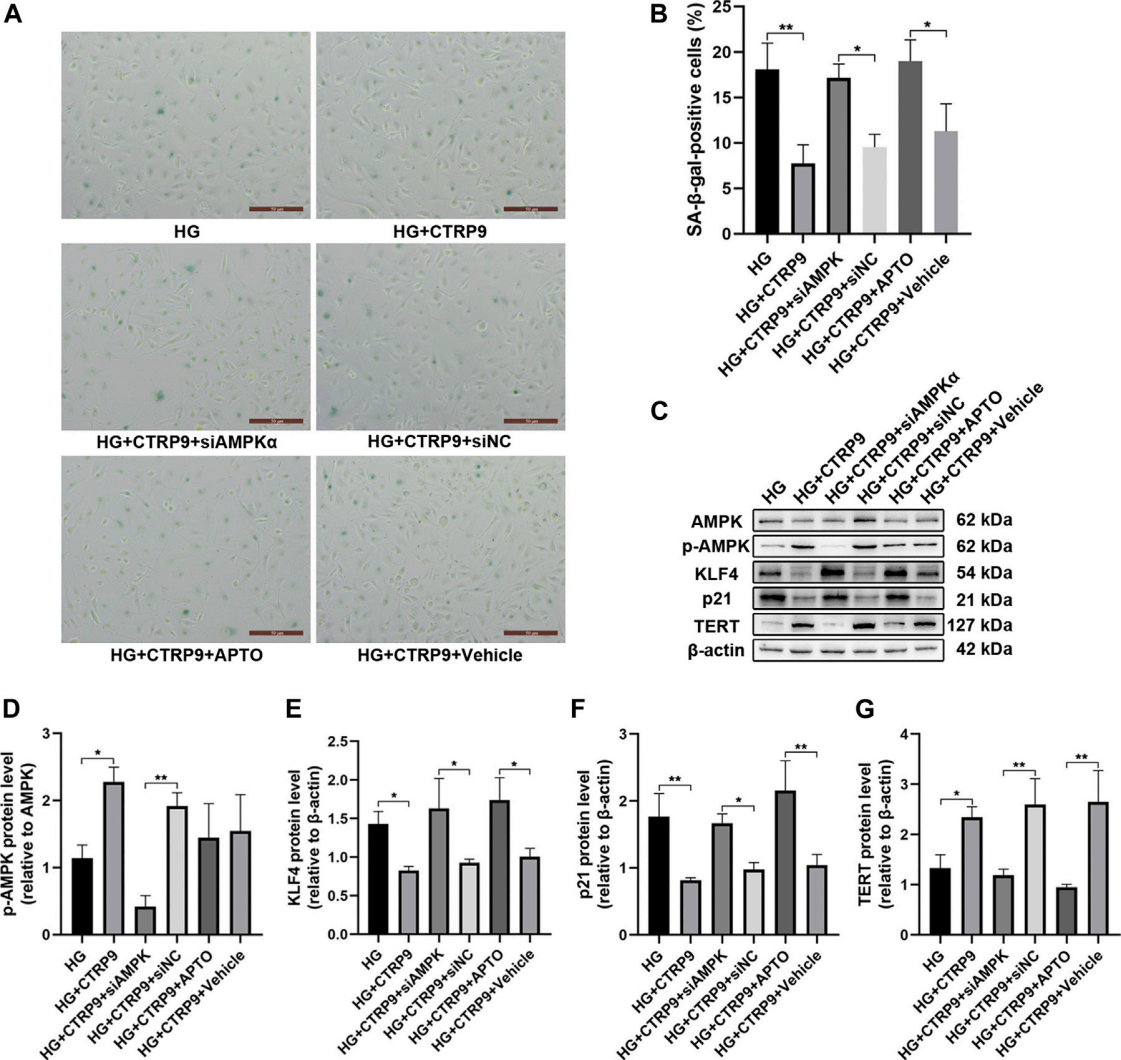
CTRP9-mediated anti-senescence effect is mediated *via* the AMPKα/KLF4 pathway. **(A)** Cells were fixed and stained for SA-β-gal activity. The images were taken at ×10magnification. **(B)** The histogram represents the quantitative analysis of SA-β-gal-positive cells per microscopic field. Values represent mean ± SEM. *p < 0.05, **p < 0.01. **(C)** AMPK, p-AMPK, KLF4, p21 and TERT protein levels were determined by immunoblotting. **(D-G)** Results were normalized to controls, and histograms represent the relative intensities of p-AMPK, KLF4, p21, and TERT. Values represent mean ± SEM (*n* = 3–4 per group). *p < 0.05, **p < 0.01.

The anti-senescence effect of CTRP9 in AMPKα-knocked down and KLF4-activated HUVECs was investigated under HG conditions. The number of SA-β-gal-positive cells was increased following AMPKα knockdown and KLF4 activation, despite treatment with CTRP9 (both *p* < 0.05, Figures 5A,B). Immunoblotting data also provided molecular evidence for the AMPKα/KLF4-dependent action of CTRP9. Thus, there was a decreased expression of KLF4 and p21 (*p* < 0.05 and <0.01, Figures 5E,F) and increased expression of p-AMPK and TERT (both *p* < 0.05, Figures 5D,G) when HUVECs were maintained with HG and treated with CTRP9. However, AMPKα-knocked down or KLF4-activated HUVECs maintained with HG exhibited a reversal of this effect (Figure 5C). Collectively, the results suggested that CTRP9-associated reduction in HG-induced endothelial senescence was mediated via an AMPKα/KLF4-dependent mechanism.

### C1q/Tumor Necrosis Factor-Related Protein 9 Treatment Inhibits HG-Induced Vascular Senescence and Reduces Atherosclerotic Plaque Formation *In Vivo*

**FIGURE 6:**
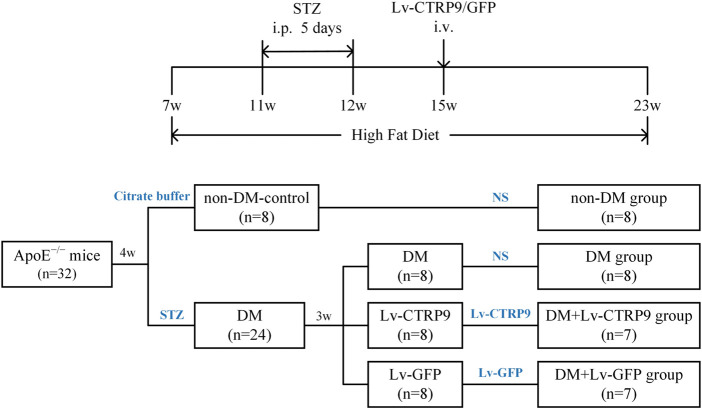
Flow chart showing the animal groups and time lines of the experimental protocol *in vivo*. DM, diabetes mellitus; STZ, streptozotocin; Lv-CTRP9, lentiviruses-mediated CTRP9 overexpressing; Lv-GFP, lentiviruses-mediated green fluorescent protein; NS, normal saline; i. p., intraperitoneal injection; i. v., intravenous injection; w, week old.

**FIGURE 7:**
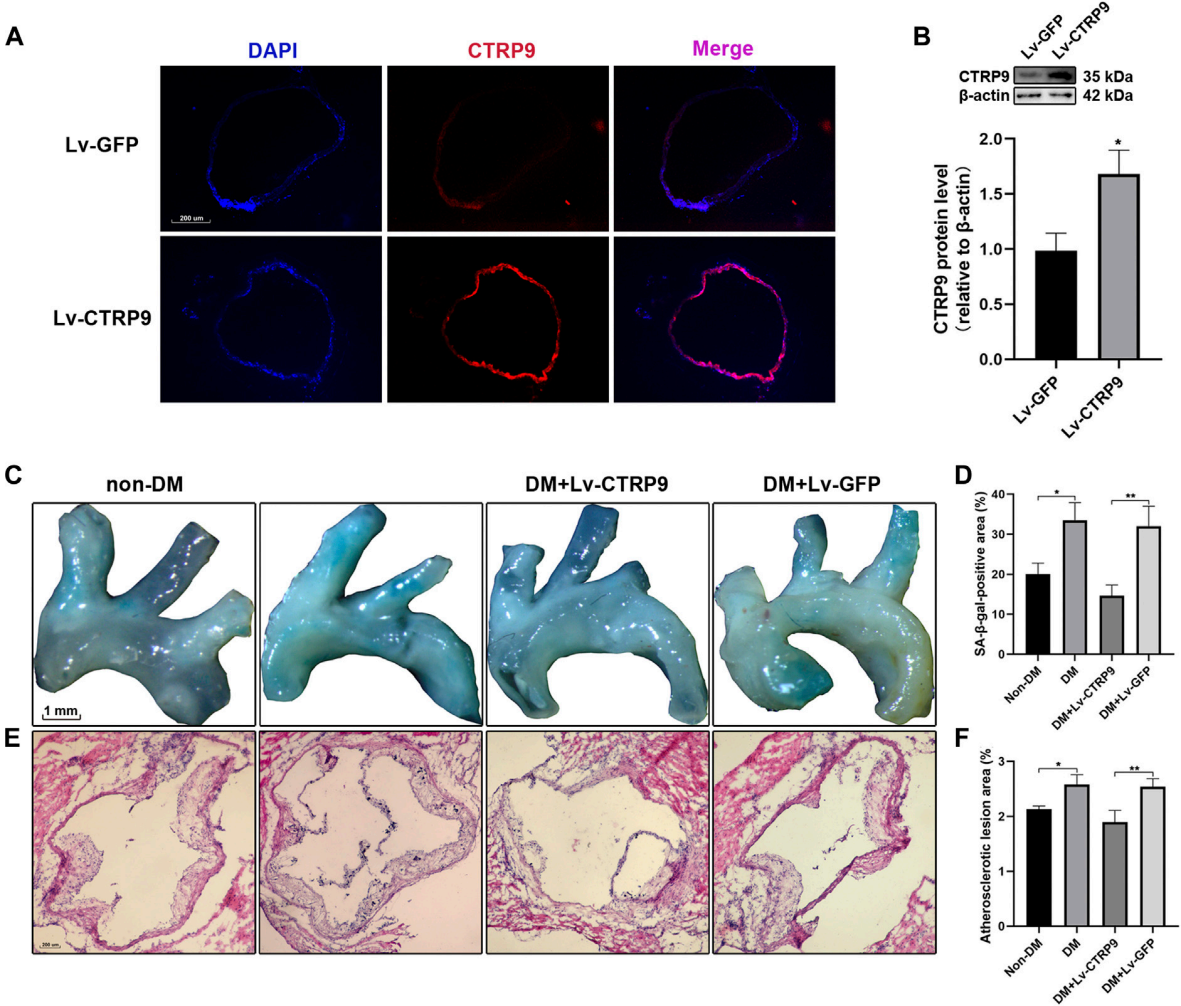
Overexpression of CTRP9 inhibits vascular senescence and reduces atherosclerotic plaque formation in STZ-induced ApoE KO mice. **(A)** Transfection efficiency of CTRP9 lentivirus in aortic roots was measured by immunofluorescence staining. The images were taken at ×100magnification and sections were stained with CTRP9 (red) and DAPI (blue). A representative image from three separate experiments is illustrated. **(B)** Expression of CTRP9 protein levels in aortic tissue was detected by immunoblotting. Results were normalized to controls, and histograms represent the relative intensity of CTRP9. Values represent mean ± SEM (*n* = 3–4 per group). *p < 0.05, significantly different from the Lv-GFP group. **(C)** The aortas were stained with SA-β-gal to analyse vascular senescence degree. **(E)** The aortas roots were stained with H&E to estimate the size of atherosclerosis lesions. **(D,F)** Quantitative analysis of lesion areas in aortas and aortic roots. Values represent mean ± SEM (*n* = 3–4 per group). *p < 0.05, **p < 0.01.

To determine the anti-senescence effect of CTRP9 in the context of atherosclerotic plaque formation, Lv-CTRP9 and Lv-GFP were injected via the tail vein into ApoE KO mice with STZ-induced diabetes (Figure 6). Immunofluorescence staining and immunoblotting showed that CTRP9 was overexpressed in the Lv-CTRP9 group (Figures 7A,B). We next used SA-β-gal and H&E staining to evaluate vascular senescence and atherosclerotic lesions. As observed with SA-β-gal staining, vascular tissues obtained from the DM group showed significantly higher SA-β-gal-positive area than did those obtained from the non-DM group (*p* < 0.05, Figures 7C,D). In addition, the DM + Lv-CTRP9 group showed decreased SA-β-gal-positive area compared with the DM + Lv-GFP group (*p* < 0.01, Figures 7C,D). H&E staining was used to estimate the size of the atherosclerotic lesions. Atherosclerotic lesions of the aortic roots were remarkably increased in the DM group compared with those in the non-DM group (*p* < 0.05, Figures 7E,F), but decreased in the DM + Lv-CTRP9 group compared with those in the DM + Lv-GFP group (*p* < 0.01, Figures 7E,F). Collectively, the *in-vitro* and *in-vivo* results supported the hypothesis that overexpression of CTRP9 inhibits cellular and vascular senescence, thereby inhibiting the progression of atherosclerosis.

## Discussion

Atherosclerosis remains a major cause of morbidity and mortality worldwide, and a thorough understanding of the underlying pathophysiological mechanisms would be crucial for the development of new therapeutic strategies (Lawler et al., 2020). Diabetes mellitus, characterized by chronic hyperglycemia, is a major risk factor for atherosclerosis. Data from human and animal studies have supported a direct proatherogenic role of hyperglycemia, particularly at the level of the arterial endothelium (Bornfeldt and Tabas, 2011). Since atherosclerosis has a distinct age dependence, its association with senescence has been clear from the very beginning. Pathologically, although atherosclerosis is initiated by the deposition of cholesterol-rich lipoproteins in the artery wall, hyperglycemia-induced endothelial premature senescence is believed to be associated with the pathogenesis of atherosclerosis (Burton and Faragher, 2018). SASP and dysfunctional cell accumulation caused by endothelial cell senescence fuel atherosclerosis progression and impair resolution (Tian and Li, 2014). Therapeutic strategies that safely and effectively inhibit endothelial senescence and interfere with the detrimental effects of endothelial senescence have currently been gaining significant attention. CTRP9 was initially discovered in adipocytes during efforts to identify paralogs of adiponectin—the most extensively investigated adipokine—and is a well-known cardiovascular protective factor (Yamauchi and Kadowaki, 2013). Previous studies have shown CTRP9 to have potent anti-diabetic, anti-oxidative, and anti-inflammatory properties, being involved in multiple pathophysiological processes associated with atherosclerosis (Yu et al., 2018). A recent study has shown that CTRP9, as an emerging potential anti-senescence molecule, ameliorates cellular senescence via AMPK signaling in mesenchymal stem cells (Li et al., 2018; Rezabakhsh et al., 2020). The finding suggested the anti-atherogenic action of CTRP9 to be possibly mediated by its anti-senescence features in the pathogenesis of atherosclerosis.

Data from the current study support the hypothesis that incubation of HUVECs in HG medium, as a model of hyperglycemia, promotes HUVEC senescence by increasing the expression of the KLF4 protein and downstream signaling targets. CTRP9, at a concentration of 3 μg/ml, activated AMPKα and reduced the expression of KLF4 to inhibit HG-induced HUVEC senescence. Furthermore, the anti-senescence effect of CTRP9 on HUVECs was lost when the expression of AMPK was silenced using an siRNA protocol or when the expression of KLF4 was induced by APTO. In addition, CTRP9 inhibited hyperglycemia-induced vascular senescence and reduced atherosclerotic plaque formation in ApoE KO mice with STZ-induced diabetes.

Previous studies have shown that the HG levels associated with diabetes promote endothelial cell senescence *in vitro* (Yokoi et al., 2006; Shosha et al., 2018). As described by Hayflick for the first time, cellular senescence occurs *in vitro* due to the finite capacity of cells to replicate (Hayflick, 1965). However, senescence can occur independent of continuous cell replication. This condition is referred to as stress-induced premature senescence, and it involves hyperglycemia (Kural et al., 2016). KLF4, a DNA-binding transcriptional regulator, controls a large number of key cellular processes and regulates cell senescence and survival in response to cellular stress. Upregulation of KLF4 typically activates CDKN1A, which encodes the cyclin-dependent kinase inhibitor p21, to promote cell cycle arrest (Shum et al., 2013). In this study, we explored the effect of KLF4 and downstream signaling targets in a HG-induced HUVECs senescence model and found HG to significantly increase the expression of KLF4 protein and that of its downstream target p21. TERT, the most essential protein subunit of telomerase, is one of the major targets of KLF4 (Yadav et al., 2019). However, we found that the HG-induced increased expression of KLF4 significantly decreased the expression of TERT in HUVECs, which is inconsistent with previous observations in stem cells and cancer cells (Wong et al., 2010; Hsieh et al., 2017). We speculated that KLF4, as a suppressor or activator, may have a context-dependent function, depending on the tissue or cell type. In the present study, the effects of HG levels were not mimicked by the osmotic control of glucose and mannitol. Thus, endothelial cell uptake and glucose metabolism are prerequisites for HG-induced endothelial senescence. Our novel findings may provide new molecular insights into the regulation of endothelial senescence.

CTRP9 has attracted considerable attention since its discovery in 2009 (Wong et al., 2009). Previous studies had shown that CTRP9 attenuates atherosclerosis by inhibiting inflammatory responses and endothelial dysfunction (Sun et al., 2017; Zhang et al., 2019). A recent study indicated that CTRP9 is an emerging potential anti-senescence molecule that plays a critical role in ameliorating cellular senescence (Li et al., 2018; Rezabakhsh et al., 2020). In the present study, we explored the pharmacological action of CTRP9 in an HG-induced HUVECs senescence model and found that treatment with CTRP9 significantly reduced the number of SA-β-gal-positive cells. Thus, the data provided evidence that KLF4 may be a key target for the anti-senescence effect of CTRP9 in an HG environment. We found treatment with CTRP9 to significantly decrease the HG-induced KLF4 protein expression. Simultaneously, in HUVECs expressing KLF4 induced by APTO, a higher β-galactosidase activity was observed, and CTRP9 treatment in these cells was no longer effective. Our results indicated that CTRP9 mitigates endothelial cell senescence by influencing the expression of KLF4. CTRP9 has been shown to improve endothelial function and protect the macro- and microvasculature in diabetes via mechanisms that appear to be independent of its hypoglycemic actions (Cheng et al., 2016; Li et al., 2019; Hu et al., 2020). Although blood sugar levels of some patients with diabetes are well controlled, recent evidence indicates that transient hyperglycemia could potentiate persistent diabetic vascular complications and induce sustained endothelial senescence, a phenomenon known as “metabolic memory” (Zhang et al., 2015). Interestingly, our study confirmed that senescence is decreased in HUVECs maintained in NG and treated with CTRP9. This finding indicated that CTRP9 has great promise in transforming the treatment of diabetes-associated atherosclerosis by improving cardiovascular outcomes independent of glycemic control, which is inconsistent with previous observations.

AMPK, a serine/threonine kinase sensitive to cellular energy alterations and processes linking energetics to longevity, acts as a major downstream component of CTRP9 signaling. CTRP9 overexpression has been reported to protect against HG-induced endothelial oxidative damage and apoptosis by phosphorylating AMPK (Cheng et al., 2016; Cheng et al., 2020). Treatment with recombinant CTRP9 protein significantly increased the phosphorylation of AMPK and inhibited cytokine-induced vascular inflammation and leukocyte adhesiveness in human aortic endothelial cells (Jung et al., 2016). Furthermore, AMPKα deficiency has been reported to promote VSMC phenotypic switch and atherosclerotic plaque instability by upregulating KLF4 (Ding et al., 2016). Considering the relationship between CTRP9 and the AMPKα/KLF4 pathway, we evaluated whether the latter was involved in HUVECs senescence inhibition by CTRP9. The effect of CTRP9 on the regulation of endothelial cell senescence in AMPKα-silenced or KLF4-activated HUVECs was investigated using HG cell culture protocols. Treatment with CTRP9 did not reduce the expression of KLF4 and p21 proteins or increase that of TERT protein in AMPKα-silenced or KLF4-activated HUVECs compared to the observation in control cells. Data from the current study supported the hypothesis that CTRP9 activates AMPKα and negatively regulates KLF4 to inhibit endothelial cell senescence. To the best of our knowledge, this is the first study to link the CTRP9-mediated AMPKα/KLF4 pathway to the effects of HG-induced HUVECs senescence. Results from the current study provide molecular insights into the cellular actions of this important cardiokine.

Senescent endothelial cells can acquire a specific phenotype, SASP. This phenotype triggers chronic inflammation and is crucial for the initiation and progression of atherosclerosis (Yin and Pickering, 2016; Shakeri et al., 2018). In this study, we explored the mRNA transcription profiles of several key SASP components in HG-induced senescence of HUVECs. We found CTRP9 treatment to significantly alter the transcription of many genes involved in inflammatory responses. Furthermore, endothelial cell dysfunction is a hallmark of vascular disease and is characterized by reduced NO bioavailability and increased ROS production. Our data suggested that CTRP9 significantly decreased the NO production and increased ROS accumulation caused by endothelial senescence. These findings extend upon previous observations (Zheng et al., 2011; Cheng et al., 2016; Li et al., 2019) and support the notion that CTRP9 treatment ameliorates the SASP and improves endothelial cell function.

Recently, CTRP9 was accepted as a cardiokine rather than adipokine, since the former has the highest expression level in an adult heart (Su et al., 2013; Appari et al., 2017). Recent animal studies have shown that CTRP9 attenuates atherosclerosis through the inflammasome and autophagy signaling pathway in ApoE KO mice (Huang et al., 2019; Zhang et al., 2019). Furthermore, CTRP9 relieved hyperglycemia-mediated oxidative stress and apoptosis of endothelial cells in diabetic db/db mice (Hu et al., 2020). In our study, we aimed to explore whether the role of CTRP9 in atherosclerotic lesion formation is exerted through its anti-senescence properties *in vivo*. We injected lentiviral vectors overexpressing CTRP9 into ApoE KO mice with STZ-induced diabetes that were fed a high-fat diet and found that overexpression of CTRP9 significantly inhibited senescence in the aorta and decreased atherosclerotic lesions. Our results indicated that CTRP9, which serves as a novel anti-senescence cardiokine, can inhibit endothelial cell and vascular senescence to delay the progression of diabetes-related atherosclerosis. Thus, designing active CTRP9 polypeptides would be a highly desirable strategy for realizing the beneficial effects of CTRP9. Very recently, utilizing I-TASSER structure prediction and 3-D active site modeling, Liu et al. identified the first CTRP9 C-terminal polypeptide (CTRP9-281), which exerts superior cardioprotective actions after myocardial infarction *in vivo* (Liu et al., 2021). This previous study confirmed and supported our view that the pharmacological effects of CTRP9 may have important transformational value in the treatment of atherosclerosis in the future. However, whether CTRP9 could also exert anti-senescence effects on senescent intimal foam cells via the activation of the AMPKα pathway remains unknown, and requires further investigatin.

## Conclusion

In conclusion, our present study provided the first evidence that hyperglycemia accelerates endothelial senescence by influencing the expression of KLF4 and its downstream signaling targets. CTRP9 mitigates endothelial cell senescence through an AMPKα/KLF4-dependent mechanism. *In-vivo* experiments showed that CTRP9 inhibited hyperglycemia-induced vascular senescence and reduced atherosclerotic plaque formation in ApoE KO mice with STZ-induced diabetes. Collectively, our novel findings demonstrated that the pharmacological action of CTRP9 may have important translational value in the treatment of type 2 diabetes mellitus‒accelerated atherosclerosis.

## Data Availability

The original contributions presented in the study are included in the article/Supplementary Material, further inquiries can be directed to the corresponding author.
