# Corrigendum: C1q/TNF-Related Protein 9 Attenuates Atherosclerosis by Inhibiting Hyperglycemia-Induced Endothelial Cell Senescence Through the AMPKα/KLF4 Signaling Pathway

**DOI:** 10.3389/fphar.2021.812384

**Published:** 2021-12-15

**Authors:** Gang Wang, Baihe Han, Ruoxi Zhang, Qi Liu, Xuedong Wang, Xingtao Huang, Dandan Liu, Weishen Qiao, Mengyue Yang, Xing Luo, Jingbo Hou, Bo Yu

**Affiliations:** ^1^ The Key Laboratory of Myocardial Ischemia Organization, Chinese Ministry of Education, Harbin, China; ^2^ Department of Cardiology Organization, The Second Affiliated Hospital of Harbin Medical University, Harbin, China; ^3^ Department of Cardiology, Harbin Yinghua Hospital, Harbin, China

**Keywords:** CTRP9, atherosclerosis, senescence, hyperglycemia, AMPK, KLF4

In the original article, there was a mistake in the artwork for Figure 2C as published. We made a careless mistake about p21 protein. The correct artwork appears below.

**FIGURE 2:**
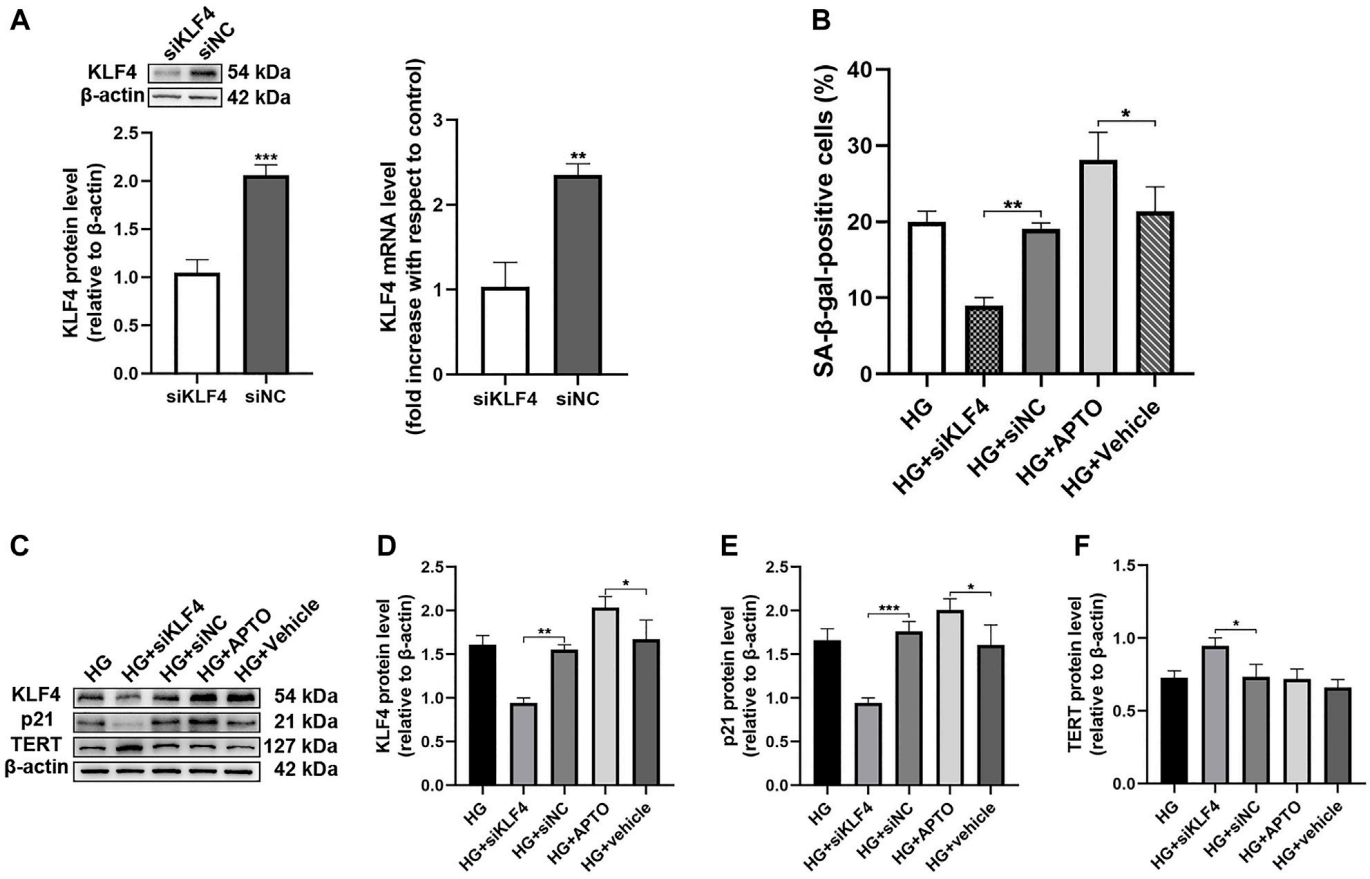
Hyperglycemia-induced HUVEC senescence is KLF4 dependent. **(A)** KLF4, as measured by immunoblotting and qRT-PCR in HUVECs after transfection with siNC or siKLF4 for 48 h. **(B)** Cells were fixed and stained for SA-β-gal activity and the histogram represents the percentage of SA-β-gal-positive cells per microscopic field. Values represent mean ± SEM. *p < 0.05, **p < 0.01. **(C)** KLF4, p21, and TERT protein levels were determined by immunoblotting. **(D–F)** Results were normalized to controls, and histograms represent the relative intensity of KLF4, p21, and TERT. Values represent mean ± SEM (n = 3–4 per group). *p < 0.05, **p < 0.01, ***p < 0.001.

The authors apologize for this error and state that this does not change the scientific conclusions of the article in any way. The original article has been updated.

