# Supplementary material for: C1q/TNF-Related Protein 9 Attenuates Atherosclerosis by Inhibiting Hyperglycemia-Induced Endothelial Cell Senescence Through the AMPKα/KLF4 Signaling Pathway
Source: Front Pharmacol. 2021 Oct 22;12:758792. doi: 10.3389/fphar.2021.758792 (PMC8569937; doi:10.3389/fphar.2021.758792)
Supplement: Supplementary file 1 [file DataSheet1.docx]

Supplementary Material

# Supplementary Figures


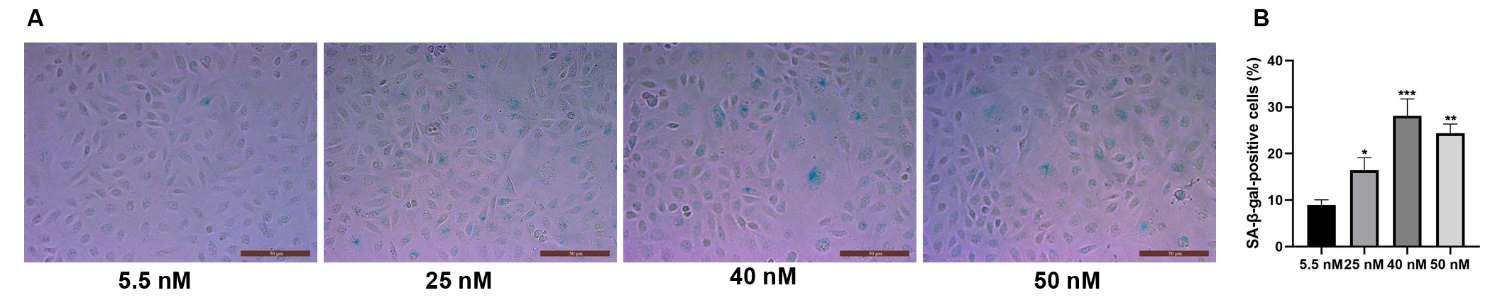


**Supplementary Figure 1.** Effect of exposure to 5.5, 25, 40, and 50 nM glucose on the senescence of HUVECs. **(A)** To determine the optimal concentration of glucose, we selected different concentrations of glucose to culture endothelial cells for 48 h. Cells were fixed and stained for SA-β-gal activity. The images were taken at 10× magnification. **(B)** Histogram represents the percentage of SA-β-gal-positive cells per microscopic field. Values represent mean ± SEM (n = 3‒4 per group). *P < 0.05, **P < 0.01, ***P < 0.001, significantly different from 5.5 nM.


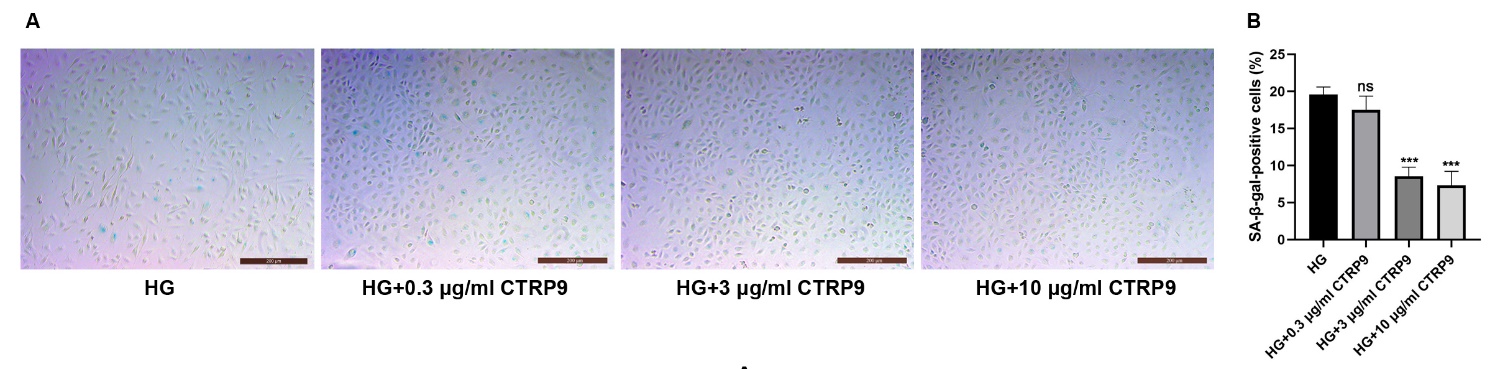


**Supplementary Figure 1.** Effect of 0.3, 3, and 10 μg/ml CTRP9 on the senescence of HUVECs induced by high glucose. **(A)** To determine the best therapeutic concentration of CTRP9, we subjected the endothelial cells cultured with high glucose to different concentrations of CTRP9. Cells were fixed and stained for SA-β-gal activity. The images were taken at 10× magnification. **(B)** Histogram represents the percentage of SA-β-gal-positive cells per microscopic field. Values represent mean ± SEM (n = 3‒4 per group). ***P < 0.001, significantly different from HG.
